# Surgical phase modelling in minimal invasive surgery

**DOI:** 10.1007/s00464-018-6417-4

**Published:** 2018-09-05

**Authors:** F. C. Meeuwsen, F. van Luyn, M. D. Blikkendaal, F. W. Jansen, J. J. van den Dobbelsteen

**Affiliations:** 1 Department of Biomechanical Engineering, Delft University of Technology, Mekelweg 2, 2628 CD Delft, The Netherlands; 2 Department of Gynecology, Leiden University Medical Center (LUMC), Albinusdreef 2, 2333 ZA Leiden, The Netherlands

**Keywords:** Workflow, Phase recognition, Patient safety, Hysterectomy, Instrument tracking

## Abstract

**Background:**

Surgical Process Modelling (SPM) offers the possibility to automatically gain insight in the surgical workflow, with the potential to improve OR logistics and surgical care. Most studies have focussed on phase recognition modelling of the laparoscopic cholecystectomy, because of its standard and frequent execution. To demonstrate the broad applicability of SPM, more diverse and complex procedures need to be studied. The aim of this study is to investigate the accuracy in which we can recognise and extract surgical phases in laparoscopic hysterectomies (LHs) with inherent variability in procedure time. To show the applicability of the approach, the model was used to automatically predict surgical end-times.

**Methods:**

A dataset of 40 video-recorded LHs was manually annotated for instrument use and divided into ten surgical phases. The use of instruments provided the feature input for building a Random Forest surgical phase recognition model that was trained to automatically recognise surgical phases. Tenfold cross-validation was performed to optimise the model for predicting the surgical end-time throughout the procedure.

**Results:**

Average surgery time is 128 ± 27 min. Large variability within specific phases is seen. Overall, the Random Forest model reaches an accuracy of 77% recognising the current phase in the procedure. Six of the phases are predicted accurately over 80% of their duration. When predicting the surgical end-time, on average an error of 16 ± 13 min is reached throughout the procedure.

**Conclusions:**

This study demonstrates an intra-operative approach to recognise surgical phases in 40 laparoscopic hysterectomy cases based on instrument usage data. The model is capable of automatic detection of surgical phases for generation of a solid prediction of the surgical end-time.

The Operating Room (OR) complex is a cost-intensive part of the hospital, as it typically accounts for more than 40% of a hospital’s total revenue and a similarly large proportion of its total expenses. Almost 60% of the patients admitted to hospitals receive operative surgical care [1]. Thus, efficient usage of OR capacity is crucial. To ensure sufficient organisational capacity, it is of utmost importance that the OR scheduling is well planned and managed timely.

Optimisation of OR scheduling is a complex task, as surgical procedure times are inherently linked to uncertainties. Various factors can alter the surgical time, such as procedure-related problems (unexpected bleeding and other adverse events) and personnel-related issues (e.g. miscommunication). However, also equipment/instrument-related issues (malfunctioning or wrong positioned) and environmental-related problems (such as disturbances by telephone or radio) are described [2].

Surgical time duration is determined by a broad range of factors such as patient characteristics, individual surgical skills and occurrence of complication. However, the current methods of OR planning are often based only on either average surgery durations or estimates by the surgical staff [3]. As both average surgery duration and estimates made by the surgical staff provide suboptimal predictive value on the real duration of the surgery, this limited approach on OR planning leads to inconsistencies between planned and actual surgery durations [4, 5]. If a procedure takes longer than scheduled, subsequent procedures have to be postponed or cancelled. On the other hand, when operations run short, the operating rooms are unutilised at the end of the day [2].

One aspect of managing OR logistics is to keep the schedule updated as the day progresses. OR schedulers typically use visual inspection to check the status of a procedure. Still, the progress is not always recognisable and one must be familiar with many procedures. An alternative is making phone calls or actually entering the OR, which is a disturbance of the surgical team. Thus, there are still major improvements to make when it comes to real-time progress monitoring.

Over the years, the interior of ORs has evolved into high-end technological masterpieces. The OR is storing a wealth of useful information through many different sources. This could range from the OR door movements and lights to the details of the anaesthetic device and the use of surgical instruments. Analysis of these data can reveal behavioural patterns, which we call the surgical workflow. With the use of intelligent algorithms, a model can be built to autonomously detect and identify different steps in the surgical procedure [6]. Through recognition of different phases during a procedure, we can also estimate how long the procedure will take and thus optimise our schedule.

Most studies have focussed on phase recognition modelling of the laparoscopic cholecystectomy, because of its standard and frequent execution [7–10]. However, to add more challenge to the phase recognition system and to extend the range of applications, more diverse and complex procedures need to be studied. By this rationale, we choose to analyse the more complex laparoscopic hysterectomy, the minimal invasive removal of the uterus. With over 600,000 hysterectomies performed yearly in the US, it is the second most common gynaecological surgical procedure [11]. Since the 1990s, a shift is seen from the traditional abdominal surgical approach to the laparoscopic or robotic one [12]. We assume this is a very suitable procedure for surgical phase recognition, due to its variability in total duration (between 98 and 214 min) [2]. The aim of this study is to find to what extent accurate phase recognition can be beneficial for long and complex procedures. Therefore, we monitor the instrument use and investigate the accuracy reached in a clinically relevant task, like surgical end-time prediction.

## Materials and methods

### Recording and transformation of surgical data

The dataset used contains 40 cases of laparoscopic hysterectomy (LH), which were recorded between November 2010 and April 2012 in the Bronovo Hospital in The Hague, The Netherlands, for the purpose of a study on surgical flow disturbances by Blikkendaal et al. [2]. The procedures were recorded using three cameras and four audio signals using an audiovisual recording system (MPEG Recorder 2.1, Noldus Information Technologies, Wageningen, The Netherlands). More detailed information about the methods used can be found in a previous publication [13].

The LH surgery was separated into 10 surgical phases and 36 surgical steps based on the method of perioperative analysis of surgeries by Den Boer et al. [2, 13], see Table 1 for a description. The phases do not necessarily occur in a chronological order. The annotated event log was exported to a plain-text file for further analysis and contained start and endpoints of all observed surgical steps, together with the 12 instruments used in predefined steps. These events represent the features used in building the surgical phase model (SPM). A single entry in the time-based log does not capture all relevant information that could be used to train the model to distinguish phases. Therefore, extra features, such as surgical time, cumulative used time of each instrument and total number of instruments currently in use, were derived from the indicators of instrument to improve the model performance. These additional data transformation and the model generation were performed using the R programming language (R Foundation for Statistical Computing, Vienna, Austria) [14] and RStudio IDE (RStudio Inc., Boston, U.S.A.) [15].

**Table 1:** Intra-operative surgical phases and steps commonly occurring during a laparoscopic hysterectomy procedure. Table copied from Blikkendaal et al. [2], based on earlier work by Den Boer et al. [13]

Phase	Step
1. Create CO_2_ pneumoperitoneum	1.1 First incision and insert Veress or Hasson
	1.2 Insufflate the abdomen
2. Insert access ports	2.1 Insert first (optical) port
	2.2 Insert laparoscope
	2.3 Inspect abdomen (active bleeding, 360 look, operatability)
	2.4 Insert second port under direct sight
	2.5 Inspect and judge operatability/unexpected pathology
	2.6 Insert third port under direct sight
	2.7 Insert fourth port under direct sight
3. Preparation operative area	3.1 Dissect adhesions to uterus/ovaria/intestine in pelvis
	3.2 Mobilise intestine out of pelvis
4. Expose uterine arteries	4.1 Dissect ligaments and mobilise uterus
	4.2 Skeletonised uterine arteries
	4.3 Push off bladder
	4.4 Identify location of ureters
5. Transect uterine arteries	5.1 Transect left uterine artery
	5.2 Transect right uterine artery
	5.3 Check colour of uterus
	5.4 Check if bladder and arteries are skeletonised enough
6. Separate uterus from vagina	6.1 Colpotomy
	6.2 Pneumoperitoneum is lost
7. Specimen retrieval	7.1 Morcellated uterus
	7.2 Extract uterus through vagina
8. Closure of the vaginal cuff	8.1 Insert needle
	8.2 Suture vaginal cuff
	8.3 Extract needle
9. Final check and irrigation	9.1 Check hemostasis
	9.2 Check vaginal cuff stump
10. Close-up patient	10.1 Remove instruments
	10.2 Remove accessory operating ports (under direct sight)
	10.3 Check access wounds/bleeding
	10.4 Release CO_2_ from abdomen
	10.5 Remove laparoscope and first trocar port
	10.6 Suture port wounds
	10.7 Remove draping

### Surgical phase modelling

For the purpose of this study, a Random Forest (RF) surgical phase recognition model was used [16]. This is an ensemble model consisting of a collection of decision trees, where each node represents a subset of the data and poses a certain question (e.g. *x* < 5). The answer to this question is used to further split the dataset and leads to another question at the following node. Finally, at the so-called leaf node, a categorical or numerical prediction of the outcome variable is obtained. Each decision tree is trained on a random subset of the training set and considers a random subset of features at each split. The prediction of each tree counts as a vote for the overall prediction. The modal (in case of classification) or mean (in case of regression) prediction of all trees provides the final prediction of the model.

### Model optimisation

An important aspect of modelling is out-of-sample validation, which involves the partitioning of the data into test and training sets. The model is generated based on the training data; validation of the model is performed on a set of unseen test data. In the current study, we use *k* fold cross-validation, in which the data are split into *k* folds, in which each acts as a single out-of-sample test set, while the model is trained on the remaining data.

Another important consideration is the choice of a performance metric for use in the out-of-sample validation. In case of a numerical prediction, a commonly reported metric is the mean absolute error (MAE). Further, at each split in the tree, a random subset of features is evaluated for deciding the best split. The number of features to select at each split is one of the most important parameters in RF. The default value for the number of selected features is ${\text{floor}}\left( {\sqrt D } \right)$, with *D* being the number of features of the object [17].

In this paper, model optimisation was performed using 10 mutually exclusive folds, each containing four surgeries. The number of features considered per split was varied with a grid search of 12 log-spaced integers between 1 and 99. During the optimisation, *n* = 100 trees were grown for each RF model. The model performance was assessed by the out-of-sample accuracy, defined as the fraction of correct predictions on an unseen set of test data.

### Surgical end-time prediction

The performance of the RF model is evaluated with respect to a relevant task in clinical practice in the OR: the prediction of surgical end-times. This refers to the number of minutes that the prediction is off compared to the real duration of the surgery. For this, a second model is obtained that uses the phase predictions to estimate the remaining surgical time. The end-time prediction is given by a multiple linear regression model using the elapsed surgical time, the phase, the number of seconds that the surgery has been in that phase and the interaction terms between phase and seconds in phase as independent variables. The mean absolute error (MAE) in the end-time prediction was also calculated.

## Results

### Laparoscopic hysterectomy

**Fig. 1:**
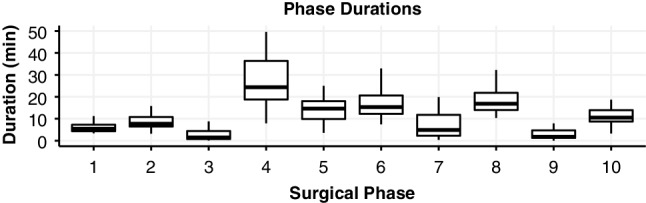
The duration of surgical phases is different per phase, but also varies strongly between procedures. The fourth phase, exposing the uterine arteries, takes the longest time to complete on average (29 min ± 13 min SD), whereas the ninth phase—final check and irrigation—has the shortest time span (3 min ± 3 min SD)

**Fig. 2:**
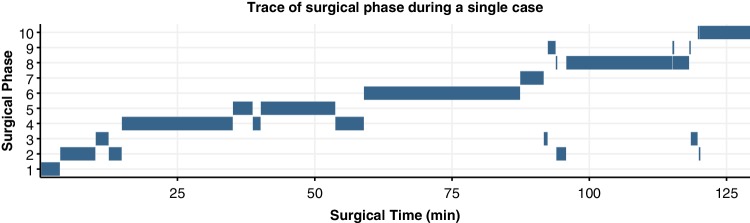
Progression of the surgical phase during a representative laparoscopic hysterectomy case. The shown case has a median case duration (129 min) and features 22 phase transitions, which is slightly above the average of 19

The analysed laparoscopic hysterectomies (*n* = 40) had an average surgery time of 128 min (± 27 min SD), with the individual surgical phases also showing a high variance in duration between cases (Fig. 1). In 33 of the LH cases, all ten phases occurred. The preparation of the operative area (phase 3) was omitted in seven cases, the closure of the vaginal cuff (phase 8) was not annotated in two cases. Although each surgery started in the first phase and ended in the last phase, phase transitions occurred 19 (± 6 SD) times per procedure on average. Most transitions, 70%, were between adjacent states, such as a transition from state one to state two. During all procedures, 68% of the state transitions were towards higher phases. A trace of the surgical phase during a representative case is shown in Fig. 2.

### Instrument use

**Fig. 3:**
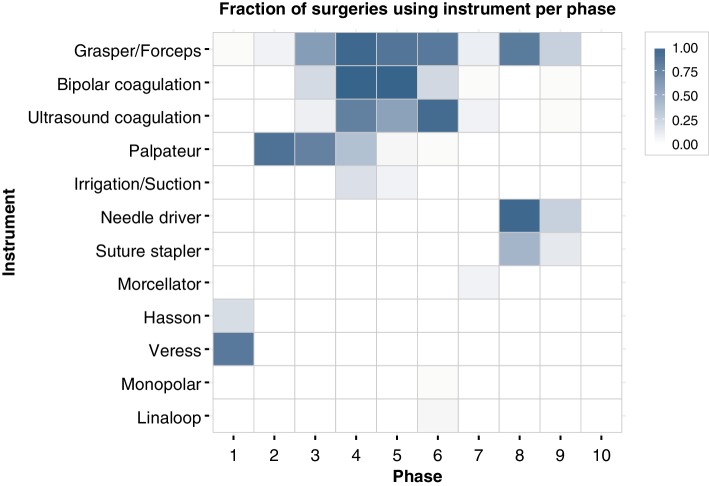
Heat map showing the frequency of instrument use per surgical phase. The fraction indicates the share of procedures during which the instrument or tool was used in the specified phase, with one indicating use in all forty LH cases. Grasper/Forceps are observed in nine out of ten phases, while the morcellator, Hasson cannula, Veress needle, monopolar coagulation and monopolar loop are only used in a single phase

The patterns of used instruments and devices differ per surgical phase (Fig. 3). With nine different phases, the grasper and forceps are most broadly used throughout the surgery, followed by the bipolar and ultrasound coagulation tools, which were both observed in six distinct surgical phases. Five tools and devices were exclusively used in one phase: the Hasson trocar and Veress needle (phase 1), the monopolar coagulation device and monopolar loop (phase 6) and the morcellator (phase 7). Some tools are observed systematically across different cases: the bipolar coagulation device is used in phase 4 and 5 in all 40 cases, the grasper/forceps in 39 cases during the fourth phase, the needle driver in 37 cases during phase 8 and the ultrasound coagulation device in 38 cases during phase 6.

### Model optimisation

**Fig. 4:**
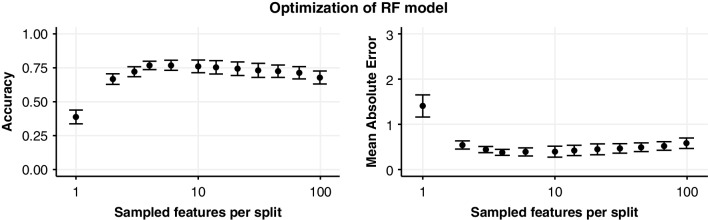
Optimisation of the RF model using 10-fold cross-validation on a grid search of 12 log-spaced parameters ranging from 1 to 98. Error bars indicate 95% confidence interval of the mean

The RF model was optimised by varying the number of evaluated features per split (Fig. 4). The ideal value was found to be 6 randomly sampled features, providing an accuracy of 76.8% (± 5.2% SD) and a mean absolute error of 0.39 phase (± 0.13 phase SD).

**Fig. 5:**
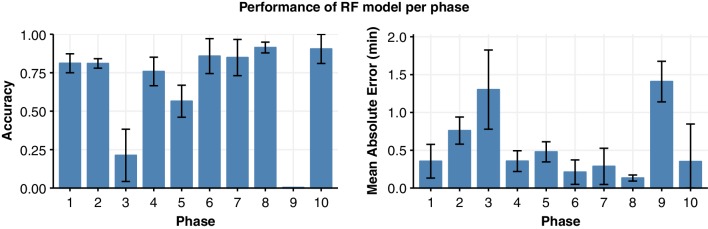
The performance of the optimised Random Forest model differs visibly per phase, ranging from 91% accuracy in phase 8 to 0.03% in phase 9. The accuracy and mean absolute error measures of model performance are strongly correlated (r = − 0.93). Error bars indicate 95% confidence interval of the mean

The overall accuracy of the model was shown to be 76.8%; however, the performance differs per phase (Fig. 5). Six of the phases are predicted accurately over 80% of their duration; phase 1 (81%), phase 2 (81%), phase 6 (86%), phase 7 (85%), phase 8 (91%), phase 10 (90%). The performance in phase 9 is lowest with an error rate of 99.7%. Again, the MAE is shown to be strongly correlated to the accuracy (*r* = − 0.93), and hence shows a similar performance pattern across the different phases.

### Surgical end-time prediction

The model performance was evaluated by application to a clinically relevant task: surgical end-time prediction. The multiple linear regression model predicts the surgical time left as the dependent variable, using surgical time passed, phase, duration within the phase and the cross terms between the phase and duration within the phase. Using ground-truth phases, we obtained a mean absolute error of 16.2 min (± 14.2 min SD) over all cases. For the regression model based on the RF-predicted phases, a MAE of 15.6 min (± 12.9 min SD) was found. Two hours before the end of the surgery, the end-time is predicted with an MAE = 17.8 min (± 14.9 min SD). This error stays rather constant for 60 min (MAE = 16.0 ± 14.0 min SD) and 45 min (MAE = 17.4 ± 11.7 min SD). At 30 min before the end of the surgery, the error drops to MAE = 12.6 ± 13.2 min SD.

## Discussion

This study demonstrates an intraoperative approach to recognise surgical phases in 40 laparoscopic hysterectomy cases based on manually annotated instrument usage data, with application to surgical end-time prediction and surgical phase extraction. The accuracy of phase detection is 77%. The performance differs per phase, ranging from 91 to 0.03%. Large variability in duration is seen between phases. For example, the phase in which the uterine arteries are exposed takes 29 min ± 13 min SD. Evaluation of the end-time prediction task shows an MAE of 15.6 min (± 12.9 min SD), which means that throughout the procedure the end-time can be calculated with an error of roughly 16 min.

In this study, we found major differences in the variability of the duration of the various phases. A high variability of a phase has a high influence on the total procedure time. Therefore, when this subset of phases has passed, the procedural time can be calculated most accurately. In this dataset phases 4, 6 and 10 are the most variable and have the most influence on the total surgical time. Detection of these phases is of utmost importance for accurate end-time prediction. Phase 9 is short in time and is the least variable. In that sense, the low accuracy of detection is not of clinical relevance.

The current study features ten surgical phases, which is higher than the number of phases observed in previous literature and as such renders the classification task more challenging, which was exactly the goal of this study. Still, the accuracy of 77% is in the range of previous findings on phase recognition using RF models (69–84%) [10, 18, 19]. Further, previous literature predicting end-times reported an MAE of 10 min [20] and 20 min [21], which is in line with our findings. However, a direct comparison is not possible due to the large differences in used data and approaches, as these previous results use either pre-operative data [20] or sensor-based recordings [21].

A major limitation of this study is the use of manually annotated data of video recordings, which cannot be used for real-time phase recognition. To further implement this technology, real-time sensor data have to be acquired. For example, promising steps have been made with the acquisition of real-time data on instrument use with an RFID-based tracking system [22–24]. Sensor data are often subject to noise, which may affect the accuracy of the model output. However, RF models have shown to be robust against noise. Also, their high computational speed is an advantage when considering the use of SPM in real time [16].

We conclude that a phase recognition model, based on the Random Forest method, shows promising accuracy to support OR planning and workflow management. Moreover, we show that tracking instruments only is sufficient to generate viable results. This study has paved the way to in vivo application of intraoperative monitoring of surgical progress.
